# Synthesis of Nylon 6,6 with Pyrene Chain-End for Compatibilization with Graphite and Enhancement of Thermal and Mechanical Properties

**DOI:** 10.3390/polym17131735

**Published:** 2025-06-22

**Authors:** Veronica Balzano, Annaluisa Mariconda, Maria Rosaria Acocella, Marialuigia Raimondo, Assunta D’Amato, Pasquale Longo, Liberata Guadagno, Raffaele Longo

**Affiliations:** 1 Department of Chemistry and Biology, University of Salerno, Via Giovanni Paolo II, 132, 84084 Fisciano, Italy; vbalzano@unisa.it (V.B.); macocella@unisa.it (M.R.A.); asdamato@unisa.it (A.D.); plongo@unisa.it (P.L.); 2 Department of Basic and Applied Sciences, University of Basilicata, Via Dell’Ateneo Lucano 10, 85100 Potenza, Italy; annaluisa.mariconda@unibas.it; 3 Department of Industrial Engineering, University of Salerno, Via Giovanni Paolo II, 132, 84084 Fisciano, Italy; mraimondo@unisa.it (M.R.); lguadagno@unisa.it (L.G.)

**Keywords:** thermoplastic composite, graphite, polyamide, compatibilization

## Abstract

The possibility of reinforcing polymeric matrices with multifunctional fillers for improving structural and functional properties is widely exploited. The compatibility between the filler and the polymeric matrix is crucial, especially for high filler content. In this paper, polymeric matrices of Nylon 6,6 with pyrene chains were successfully synthesized to improve the compatibility with carbonaceous fillers. The compatibility was proven using graphite as a carbonaceous filler. The different properties, including thermal stability, crystallinity, morphology, and local mechanical properties, have been evaluated for various filler contents, and the results have been compared to those of synthetic Nylon 6,6 without pyrene chain terminals. XRD results highlighted that the compatibilization of the composite matrix may lead to an intercalation of the polymeric chains among the graphite layers. This phenomenon leads to the protection of the polymer from thermal degradation, as highlighted by the thermogravimetric analysis (i.e., for a filler content of 20%, the beginning degradation temperature goes from 357 °C for the non-compatibilized matrix to 401 °C for the compatibilized one and the residual at 750 °C goes from 33% to 67%, respectively. A significant improvement in the interphase properties, as proven via Atomic Force Microscopy in Harmonix mode, leads to a considerable increase in local mechanical modulus values. Specifically, the compatibilization of the matrix hosting the graphite leads to a less pronounced difference in modulus values, with more frequent reinforcements that are quantitatively similar along the sample surface. This results from a significantly improved filler distribution with respect to the composite with the non-compatibilized matrix. The present study shows how the thermoplastic/filler compatibilization can sensitively enhance thermal and mechanical properties of the thermoplastic composite, widening its potential use for various high-performance applications, such as in the transport field, e.g., for automotive components (engine parts, gears, bushings, washers), and electrical and electronics applications (heat sinks, casing for electronic devices, and insulating materials).

## 1. Introduction

Nylon, obtained from polyamides (PA), is a versatile thermoplastic with many applications. It is used in the clothing industry and for producing printed and extruded products [1,2,3,4]. Nylon 6,6 (PA66), obtained by polycondensation of hexamethylenediamine with adipic acid, has excellent mechanical properties, dimensional stability, and wear resistance [5,6,7]. It is also resistant to many chemicals [8,9] and exhibits good electrical properties [10]. It is a strong and rigid thermoplastic polymer classified in the engineering plastics group, characterized by a high melting point. It is used in applications requiring high temperatures and/or chemical or abrasion resistance. Furthermore, it also exhibits low friction properties, making it ideal for electrical insulation and automotive components [11,12,13].

Continuous technological progress has led to the development of innovative materials that can ensure superior material performance and advance a green economy by conserving resources, saving time, and reducing energy costs.

In this regard, additive manufacturing has significantly altered the dynamics of today’s industrial production [14,15,16]. To increase nylon’s strength, it can be filled with glass, which makes it stronger, harder, and more rigid [17,18,19,20,21]. This also provides it with greater resistance to creep and tensile stress, improving stability when exposed to temperature fluctuations. Additionally, glass filling offers good fatigue resistance, high mechanical damping properties, improved dimensional stability, and enhanced wear resistance. Finally, it allows nylon to reach higher maximum service temperatures [22]. However, adding glass as a filler also has disadvantages, such as substantially higher costs, higher specific weight, greater fragility, and increased abrasiveness and friction [23,24,25,26]. On the other hand, many papers published in the literature over the last few decades demonstrate that nanostructured forms of carbon can impart several relevant properties to polymeric matrices hosting these kinds of fillers; among them are joule heating [27,28,29] and structural health monitoring [30,31]. Recently, Mingione et al. produced composites of polyamide filled with graphite nanoplatelets to improve the thermal, electrical, and tribological properties of the material [32].

Nylon can also be loaded with carbonaceous fillers, which gives the material an excellent weight-to-stiffness ratio and increases its lightness, strength, resistance, durability, dimensional stability, and resistance to thermal distortion [33].

Carbon-filled nylon also has excellent sliding properties and is less abrasive than glass-filled nylon. Furthermore, carbon provides superior thermal conductivity, which translates into the rapid dissipation of frictional heat, a crucial property for sliding applications (e.g., bearings) [34,35]. Thus, carbon-filled nylon is an exceptional material suitable for transverse applications, such as in the automotive and aerospace industries, for creating mobile structures and articulated objects in the medical, ornamental, and recreational fields [36]. It enables the creation of functional prototypes and end-use parts that require reinforcement, rigidity, and resistance, while maintaining a reduced density and the lowest possible weight. Enhancing the thermal and mechanical properties of these materials is crucial. Nylon is generally characterized by a narrow processing window, defined as the range between the melting point and the thermal degradation [37]. By enhancing the thermal and mechanical properties of the materials, it is possible to improve their processability and utilize them, thereby ensuring higher thermal stability and mechanical performance, parameters crucial in the transportation sector.

However, it is worth noting that combining materials with different chemical characteristics can lead to the formation of interphase regions. This is especially important in the case of carbon reinforcement, since its surface is chemically inert and hydrophobic, resulting in a weak bond between the carbon filler and the polymer matrix [38]. Furthermore, the carbon filler particles tend to agglomerate due to their high surface area and the accumulation of van der Waals forces [39]. To overcome this problem, the graphite surface can be modified to ensure better dispersion and interaction with the polymer matrix. This modification can be achieved by covalent or non-covalent functionalization. The former often destroys the conjugated structure of the graphite surface, compromising some of its properties, while the latter preserves the original structure. Among the non-covalent modifications, those involving interactions between graphite and polycyclic aromatic molecules, resulting in π-π type interactions, are particularly interesting. Synthesizing a polyamide with a pyrene terminal enables non-covalent interactions, i.e., it is reasonable to hypothesize that π-π stacking interactions can be established between the aromatic rings of graphite and those of the pyrene group of the polyamide [40,41,42,43]. Mechanical methods, such as ball milling, can overcome the weak van der Waals forces between graphite layers, causing them to separate [44]. This technique relies on a shear-force dominant process. The particle size is reduced through impact and attrition, primarily using metallic balls (typically zirconia (ZrO_2_) or steel) as the grinding media within a rotating shell. The rotation generates centrifugal force, facilitating the milling process [45]. Thus, to improve the compatibilization between the polymer matrix and the carbonaceous filler, in this work, we synthesized and fully characterized a PA66 with a pyrene group as a terminal chain (PA66py). The obtained polymer was mixed by ball milling with different percentages of graphite, and the resulting composite materials (GPA66py) were compared with analogous materials obtained using non-functionalized PA66 and graphite in the same proportions (GPA66). In general, we have demonstrated that the compatibility of the graphitic structure, enhanced by the presence of the pyrenic chain, guarantees higher thermal stability and higher local mechanical performance, as measured by Atomic Force Microscopy (AFM) utilizing the “HarmoniX” acquisition method.

## 2. Materials and Methods

All the reagents were bought from Merck Darmstadt, Germany: 1-Pyrenebutyric acid (97%), Hexamethylenediamine (98%), Adipoyl chloride (98%), graphite (C 99.8 wt%, Asbury graphite Mills Inc. 156 Asbury-West Portal Road. Asbury, NJ, USA) and methanol were used as received, whereas NaOH 1M was prepared appropriately.

All the products obtained were characterized using various techniques, including nuclear magnetic resonance and Fourier transform infrared (FTIR) spectroscopies, thermogravimetric analysis (TGA), differential scanning calorimetry (DSC), wide-angle X-ray diffraction (WAXD), and atomic force microscopy (AFM). The specifications of the instruments used are listed below.

Concerning the nuclear magnetic resonance spectroscopy, the products were characterized using Proton Nuclear Magnetic Resonance (^1^H NMR) and Carbon-13 Nuclear Magnetic Resonance (^13^C NMR) spectroscopy. The samples were prepared by dissolving approximately 10 mg of the product in 0.1 mL of hexafluoro-2-propanol and 0.4 mL of deuterated chloroform (Eurisotop Cambridge Isotope Laboratories, Cambridge, UK). For NMR analysis, a Bruker AM 600 (Milano, Italy) spectrometer (600 MHz for ^1^H; 150 MHz for ^13^C), AM 400 (Milano, Italy) spectrometer (400 MHz for ^1^H; 100 MHz for ^13^C), and AM 300 (Milano, Italy) spectrometer (300 MHz for ^1^H; 75 MHz for ^13^C) were used. The chemical shifts refer to the residual peaks of the deuterated solvent for proton (^1^H: CD_3_OD, δ = 3.31 ppm; CDCl_3_, δ = 7.26 ppm) and alcohol additive (^1^H: HFP, δ =5.22 ppm; 3.15 ppm), as well as the carbon signals (^13^C: CD_3_OD, δ = 49.00 ppm; CDCl_3_, δ = 77.16 ppm) and (^13^C: HFP, δ =120.0 ppm; 70.3 ppm). Chemical shift (ppm), multiplicity, and integration were specified for each signal from the ^1^H and ^13^C NMR spectra. Multiplicities have been abbreviated as follows: singlet (s), doublet (d), triplet (t), multiplet (m), and broad (br).

Fourier transform infrared spectroscopy (FTIR) spectra were obtained at a resolution of 2.0 cm^−1^ with an FTIR (BRUKER Vertex 70) spectrometer equipped with a deuterated triglycine sulfate (DTGS) detector and a KBr beam splitter using KBr pellets, in the wavelength range from 4000 to 400 cm^−1^, with 32 scans and a resolution of 2.0 cm^−1^.

Thermogravimetric analysis (TGA) measurements were carried out using a Q500 TA Instruments (Milano, Italy). Samples of 5 mg were placed in platinum pans, and experiments were conducted in nitrogen, from 25 °C to 800 °C, at a rate of 10 °C min.

DSC measurements were carried out using a thermal analyzer, Mettler DSC 822/400 (Mettler-Toledo), purged with nitrogen. The samples were first heated from −60 °C to 280 °C with a heating rate of 10 °C/min, then cooled to −60 °C at −10 °C/min, and then heated again from −60 °C to 280 °C with a heating rate of 10 °C/min.

Wide-angle X-ray diffraction (WAXD) patterns were obtained using an automatic Bruker D2 phaser diffractometer, in reflection, at 35 KV and 40 mA, using nickel-filtered Cu Kα radiation (1.5418 Å).

A planetary ball mill, Pulverisette 7 Premium (Fritsch GmbH, Germany), at room temperature, was used for the ball milling experiments that were conducted using a silicon nitride jar in which eight silicon nitride balls with a diameter of 10 mm were introduced.

The morphology of the samples was examined by atomic force microscopy (AFM) using a NanoScope MultiMode V scanning probe microscope (Veeco, Santa Barbara, CA, USA) fitted with a HarmoniX tool (H-AFM). Specifically, AFM, which utilizes the “HarmoniX” acquisition method, has been used to investigate both qualitative and quantitative maps of mechanical properties at the nanometric resolution, thus combining high-resolution imaging (topographic map) with the ability to measure local mechanical properties like elasticity and adhesion (mechanical map). Below it is reported how it works:

- Dynamic Oscillation: the tip of the atomic force microscope oscillates at a specific frequency while scanning the sample surface. This oscillation allows the system to detect variations in mechanical properties.
- Force-Distance Curves: as the tip interacts with the sample, it generates force–distance curves which provide information about the material’s stiffness, elasticity, and other mechanical characteristics.
- Real-Time Mapping: HarmoniX processes the data in real time to create detailed maps of mechanical properties across the sample surface, offering insights into the material’s heterogeneity.

This technology is particularly valuable for studying complex materials like polymers and composites [46,47,48].

Tests were conducted using HMX probe silicon cantilevers (Bruker, Billerica, MA, USA) with a nominal radius of approximately 10 nm. The cantilever oscillation consists of two distinct movements: vertical and torsional. These movements exhibit varying frequencies; specifically, the amplitude frequency of the torsional movement exceeds the tapping frequency [49,50].

The reconstruction of sample morphology results from vertical movements in the standard tapping mode, while the elastic modulus maps are reconstructed due to the interactions between the tip and sample forces during torsional motion [49]. HarmoniX measurements were conducted in the air. Cantilevers were adjusted using a typical polystyrene/low-density polyethylene (PS/LDPE) specimen. The selected vertical frequency was 49 kHz, while the torsional frequency measured 1044 kHz. Imaging was conducted at a scan rate of 0.5 Hz, taking into account 20 harmonics. The four images for Height, Phase, Peak Force, and Average Force were shown for each analyzed sample. These images, examined using NanoScope Analysis version 1.80, are provided simultaneously because they collectively offer a comprehensive understanding of the surface and material properties at the nanoscale. In particular, the Height image represents the topographical information of the sample being scanned. It is a map of the surface’s height variations, showcasing peaks, valleys, and overall roughness. This image is crucial for analyzing the surface morphology and is typically generated by the AFM probe’s interaction with the sample, detecting vertical movements as it scans. The Phase image represents the phase shift between the oscillation of the AFM probe and the driving signal during its interaction with the sample. Phase imaging is particularly useful for distinguishing between areas of different material composition on a heterogeneous sample. In HarmoniX mode, the Phase image can complement outputs like the Height image, offering a deeper understanding of the sample’s mechanical and chemical characteristics. The Peak Force image represents the mapping of the peak interaction forces between the AFM probe and the sample surface during scanning. This image visualizes the force exerted by the tip at each point on the surface.

The Peak Force image is frequently used for studying mechanical properties, such as stiffness or adhesion, as it highlights variations in material interactions across the sample. It complements other images, such as Height and Phase, by providing specific insights into the force dynamics at play.

The Average Force image represents the mapping of the mean force exerted by the AFM probe as it interacts with the sample surface during scanning. This image provides a quantitative overview of the sustained interaction forces across different regions of the sample.

The Average Force can be linked to material properties such as stiffness or adhesion, and it complements other outputs, like peak force or height images, by offering a more balanced measure of force interaction over time. It is particularly valuable for studying samples with varying mechanical properties or heterogeneous compositions.

### 2.1. Synthesis of N-(6-Aminohexyl)-4-(Pyren-1-yl)Butanamide (A6py)

In a two-neck round-bottom flask, equipped with a magnetic stirrer and a cooler under an inert atmosphere, hexamethylenediamine (0.201 g, 1.73 mmol) and 25 mL of methanol were introduced. The mixture was stirred for 10 min at room temperature. Then, using a dropping funnel, a solution of 1-pyrenebutyric acid (0.250 g, 0.87 mmol) dissolved in the remaining 25 mL of methanol was added dropwise to the mixture. The system was stirred at room temperature for 5 h. Afterward, the product was recovered by removing the solvent using a rotary evaporator, yielding a yellow solid. Yield: 78%.

^1^H NMR (300 MHz, CD_3_OD) δ: 8.39–7.91-(8H, aromatics); 3.39 (t, 2H, J 8.10 Hz, (C*H*_2_(C=O)NH)); 2.79 (t, 4H, J 7.63, Hz, (NH-C*H*_2_-(CH_2_)_4_-C*H*_2_-NH_2_)); 2.37 (t, 2H, J 7.29 Hz, (Ar-C*H*_2_)); 2.15 (m, 2H, (Ar-CH_2_-C*H*_2_)); 1.58–1.37 (br s, 8H, (NH-CH_2_-(C*H*_2_)_4_-CH_2_-NH_2_)).

^13^C NMR (75 MHz, CD_3_OD) δ: 182.2_9_ (CH_2_(*C*=O)NH); 138.0_9_ -124.5_3_ (aromatics); 41.2_9_ (NH-*C*H_2_-CH_2_-(CH_2_)_2_-CH_2_-*C*H_2_-NH_2_); 38.9_0_ (*C*H_2_(C=O)NH); 34.2_2_ (Ar-*C*H_2_); 30.9_4_ (NH-CH_2_-*C*H_2_-(CH_2_)_2_-*C*H_2_-CH_2_-NH_2_); 29.9_0_ (NH-CH_2_-CH_2_-(*C*H_2_)_2_-CH_2_-CH_2_-NH_2_); 27.1_4_ (Ar-CH_2_-*C*H_2_). FTIR (KBr, cm^−1^) 1638_ν(C=O)_; 1548_ν(C-N)_; 1044 and 833_ν(C-H)_.

### 2.2. Synthesis of N-(6-Aminohexyl)-4-(Pyren-1-yl)Butanamide-Nylon 6,6 (PA66py)

The polymer was prepared via interfacial polycondensation. In the first beaker, the aqueous phase was prepared by adding 25 mL of water and NaOH 1M (3.1 mL). Then, A6py (0.044 g, 0.13 mmol) was added and stirred until completely dissolved, followed by the addition of hexamethylenediamine (0.747 g, 6.4 mmol). The A6py:hexamethylenediamine ratio was (1:50). In a second beaker, the organic phase was prepared by adding 25 mL of cyclohexane and adipoyl chloride (0.630 g, 3.4 mmol), with an adipoyl chloride– hexamethylenediamine ratio of (1:1.9). The polymer is formed by slowly adding the contents of the organic solution to the beaker containing the aqueous solution using a Pasteur pipette. The formation of the polymer at the interface is observed, and using an iron wire, it is drawn and wound onto a glass rod. The polymer is then thoroughly washed with water and left to dry in a *vacuum* oven at 50 °C overnight.

^1^H NMR: (400 MHz, HFP/CDCl_3_) δ: 8.29–7.87 (aromatics); 6.34 (br, (-N*H_2_*)); 5.44 (br, (-N*H*-CH_2_)); 3.22 (br s, overlapping, 6H, ((C=O)C*H*_2_-(CH_2_)_2_-C*H*_2_(C=O)), (C*H*_2_(C=O))); 2.54 (br s, overlapping, 2H, (Ar-C*H*_2_)); 2.41(br s, overlapping, 6H, ((C=O)CH_2_-(C*H*_2_)_2_-CH_2_(C=O)); 2.25, (br s, overlapping, 8H, ((NH-C*H*_2_-(CH_2_)_4_-C*H*_2_-NH_2_)); 1.67–1.33 (br s, overlapping, 16H, (NH-CH_2_-(C*H*_2_)_4_-CH_2_-NH_2_)).

^13^C NMR: (150 MHz, HFP/CDCl_3_) δ: 177.8_8_ (CH_2_(*C*=O)NH); 135.4_9_ -123.8_7_ (aromatics); 40.2_6_ (NH-*C*H_2_-CH_2_-(CH_2_)_2_-CH_2_-*C*H_2_-NH_2_); 33.1_4_ (Ar-*C*H_2_); 35.6_1_ (*C*H_2_(C=O)NH, (C=O)*C*H_2_-(CH_2_)_2_-*C*H_2_(C=O)); 28.5_8_ (NH-CH_2_-*C*H_2_-(CH_2_)_2_-*C*H_2_-CH_2_-NH_2_); 26.1_1_ (NH-CH_2_-CH_2_-(*C*H_2_)_2_-CH_2_-CH_2_-NH_2_); 24.9_0_ (C=O)CH_2_-(*C*H_2_)_2_-CH_2_(C=O); 23.9_8_ (Ar-CH_2_-*C*H_2_). FTIR (KBr, cm^−1^) 1639_ν(C=O)_; 1546_ν(C-N)_; 1384_ν(C-H)_; 1044 and 833_ν(C-H)_.

### 2.3. Synthesis of Nylon 6,6 (PA66)

The polymer was prepared via interfacial polycondensation. In the first beaker, the aqueous solution was prepared by adding 25 mL of water, 3.1 mL of NaOH 1 M, and 0.75 g (6.4 mmol) of hexamethylenediamine.

In the second beaker, the organic phase was prepared by adding 25 mL of cyclohexane and adipoyl chloride (0.630 g, 3.4 mmol). The adipoyl chloride–hexamethylenediamine ratio was 1:1.9.

The polymer is formed by slowly adding the organic solution to the beaker containing the aqueous solution using a Pasteur pipette. The formation of the polymer at the interface is observed, and using an iron wire, it is drawn and wound onto a glass rod. The polymer is then thoroughly washed with water and left to dry in a vacuum oven at 50 °C overnight.

^1^H NMR: (400 MHz, HFP/CDCl_3_) δ: 6.22 (br, (-N*H_2_*)); 5.49 (br, (-N*H*-CH_2_)); 3.23 (br, s, overlapping, 4H, (C=O)C*H*_2_-(CH_2_)_2_-C*H*_2_(C=O)); 2.53 (br, s, overlapping, 4H, (NH-C*H*_2_-(CH_2_)_4_-C*H*_2_-NH_2_)); 2.25 (br, s, overlapping, 4H, (C=O)CH_2_-(C*H*_2_)_2_-CH_2_(C=O)); 1.63–1.35 (br, s, overlapping, 8H, (NH-CH_2_-(C*H*_2_)_4_-CH_2_-NH_2_)).

^13^C NMR:(150 MHz, HFP/CDCl_3_) δ: 175.9_9_ (CH_2_(*C*=O)NH); 39.9_0_ (NH-*C*H_2_-CH_2_-(CH_2_)_2_-CH_2_-*C*H_2_-NH_2_); 35.7_3_ (*C*H_2_(C=O)NH); 28.5_2_ (NH-CH_2_-*C*H_2_-(CH_2_)_2_-*C*H_2_-CH_2_-NH_2_); 26.0_1_ (NH-CH_2_-CH_2_-(*C*H_2_)_2_-CH_2_-CH_2_-NH_2_); 24.9_2_ (C=O)CH_2_-(*C*H_2_)_2_-CH_2_(C=O). FTIR (KBr, cm^−1^) 2944_ν(C-H)_; 1644_ν(C=O)_; 1545_ν(N-H)_; 1387_ν(C-H)_; 1202_ν(C-N)_; 685_ν(C-H)_

### 2.4. Preparation of PA66 and PA66py Samples with Graphite

Samples graphite/PA66 and graphite/PA66py were labeled as GPA66 and GPA66py, respectively, according to the ratio between graphite and PA66 and graphite and PA66py. A total of 100 mg of several samples was prepared at three different graphite–PA66 and graphite–PA66py weight percentage ratios: 90:10, 50:50, and 20:80. All samples were prepared twice using ball milling (BM), to obtain reproducible results. Ball mill experiments were conducted using a planetary mill at room temperature in a silicon nitride jar in which eight silicon nitride balls with a diameter of 10 mm were introduced. The rotational speed of 300 rpm was set for one hour with breaks every 5 min. All the prepared samples were subjected to thermogravimetric analysis, X-ray diffraction spectroscopy, and differential scanning calorimetry (DSC), and subsequently analyzed for further characterization.

## 3. Results

### 3.1. Synthesis of Polyamide 6,6 with a Pyrene End Group (PA66py)

**Scheme 1:**
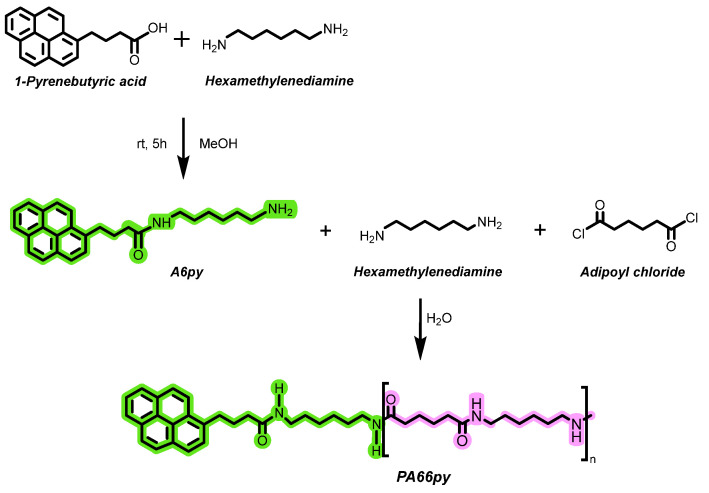
Synthesis of polyamide 6,6 with a pyrene end group (PA66py).

The synthesis of PA66py was achieved by the sequence of reactions reported in Scheme 1.

To prepare a polyamide with a pyrene group as its terminal, we proceed first by reacting the pyrene derivative (1-pyrenebutyric acid) with hexamethylene diamine. This reaction produces *N*-(6-aminohexyl)-4-(pyren-1-yl)butanamide (A6py) in high yield. The compound, A6py, is then characterized using several spectroscopic techniques: ^1^H and ^13^C NMR, FT-IR, and thermogravimetric analysis. In the ^13^C NMR spectrum, a signal at 182.2_9_ is observed, attributable to carbonyl carbon. This indicates that the reaction has occurred, as the carbonyl carbon of 1-pyrenebutyric acid typically resonates at 178.0_7_ ppm. In the FT-IR spectrum, in addition to the expected signals for the carbonyl (C=O) at 1638 cm^−1^ and the amide (C-N) at 1548 cm^−1^, there are additional signals that should be highlighted at 1051 and 844 cm^−1^, attributable to ring torsion and C-H bending of the pyrene group, respectively.

Once the terminal of the polyamide chain was obtained, the polymer chains were grown on these terminals using interfacial polymerization. This was achieved by placing hexamethylenediamine and A6py in the basic aqueous solution of NaOH, while adipoyl chloride was dissolved in the organic cyclohexane solution. The ratio of the reagents A6py:Hexamethylendiamine:Adipoyl chloride was 1:50:26. By slowly adding the organic solution to the aqueous solution, the polymer is formed at the interface. The polymer was characterized by ^1^H and ^13^C NMR, FT-IR, and thermogravimetric analysis. In the ^1^H NMR spectrum, the expected signals of polyamide, along with some resonances in the aromatic region attributable to protons bonded to aromatic carbons, were observed.

In the ^13^C NMR spectrum, in addition to the polyamide signals, some resonances in the aromatic region corresponding to aromatic carbons (about 135–123 ppm) were detectable. In the FT-IR spectrum, in addition to the expected signals of the carbonyl and the amide at 1693 cm^−1^ (C=O) and 1546 cm^−1^ (C-N), a strong signal at 1700 cm^−1^ and two weak signals at 1044 cm^−1^ and 833 cm^−1^ were observed, corresponding to ring torsion and C-H bending of the pyrene group, respectively. The slight shift in these signals in the presence of graphite may be due to the interaction of the pyrene group with the graphene planes.

### 3.2. Synthesis of Polyamide 6,6 (PA66)

**Figure 1:**
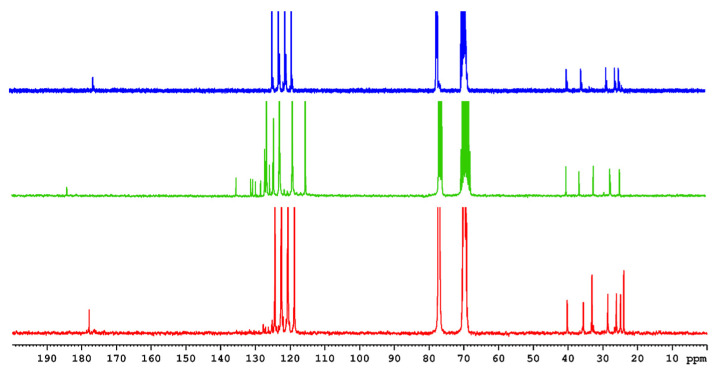
^13^C NMR spectra of PA66py (red), A6py (green), and PA66 (blue).

**Figure 2:**
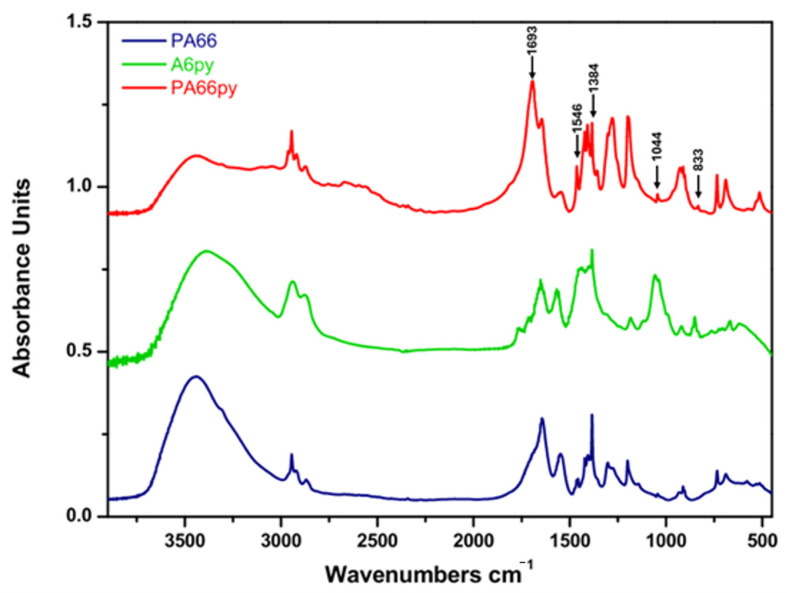
FT-IR spectra of PA66py (red), A6py (green), and PA66 (blue).

Polyamide was obtained through interfacial polycondensation by dissolving hexamethylenediamine in a NaOH solution and adipoyl chloride in cyclohexane. The molar ratio between hexamethylenediamine and adipoyl chloride was 1.9. The organic solution was slowly added to the aqueous solution, and nylon 6,6 (PA66) was formed at the interface. ^1^H- and ^13^C-NMR, as well as FT-IR spectroscopy, were used to analyze the product. To prepare the NMR sample, the polymer was initially dissolved in 1,1,1,3,3,3-hexafluoro-2-propanol (HFP), followed by the addition of chloroform-d (CDCl_3_) to achieve a final composition of 0.1:0.4 mL HFP/CDCl_3_ solvent mixture. The ^1^H and ^13^C NMR spectra [51] show methylene proton signals between 3.23 and 1.35 ppm and methylene carbon signals within the 39.9–24.9 ppm range, respectively, with a carbonyl resonance at 175.9 ppm (see Figure 1). As expected, the FTIR spectrum [18] reveals that the absorption bands corresponding to the C-H stretch are observable in the region of 2950 to 2800 cm^−1^ (see Figure 2). The absorption band associated with the stretching of the C=O group appeared at 1644 cm^−1^. This shift from the typical C=O stretching band (usually observed between 1760 and 1665 cm^−1^) can be attributed to intramolecular hydrogen bonding between the carbonyl and amino groups. Additionally, the amide II band is observed at 1545 cm^−1^, corresponding to N-H bonding. The stretching vibration of the C-N bond is evident around 1202 cm^−1^, while the deformation of the methylene group is seen at 1378 cm^−1^ and 685 cm^−1^.

### 3.3. Preparation of Graphite/PA66py Samples (GPA66py)

Approximately 100 mg of several GPA66py samples were prepared with varying weight ratios of graphite and GPA66py, specifically 90/10, 50/50, and 20/80. Each composition was obtained using the ball milling technique. All samples were analyzed by thermogravimetric analysis, differential scanning calorimetry, and X-ray diffraction spectroscopy.

### 3.4. Preparation of Graphite/PA66 Samples (GPA66)

The samples without the pyrene headgroup were also prepared using the ball milling technique to compare them usefully with those obtained by mixing graphite and GPA66py. Approximately 100 mg of each GPA66 sample was prepared with different weight ratios of graphite and PA66, namely 90/10, 50/50, and 20/80. Thermogravimetric analyses and X-ray diffraction spectroscopy were used for characterization. Note that the mixing results in the case of samples prepared with the 90/10 and 50/50 graphite/polymer ratios are represented by a flocculent fraction and a dusty fraction (reported in Table 1), both when PA66 and graphite, as well as PA66py and graphite, are subjected to ball milling. The dusty fraction is predominantly graphite. In the case of the 20/80 graphite/polymer ratio, only the flocculent fraction is obtained.

As previously reported [42,52], it is possible to assume that a small polycyclic aromatic molecule such as pyrene or benzopyrene can easily intercalate between the graphitic layers. However, if these groups are linked to polymer chains, this may not necessarily occur.

However, the presence of a pyrene group at the terminal end of a polyamide chain can facilitate the formation of numerous π-π stacking interactions, enhancing the polymer’s compatibility with the carbonaceous filler. The graphite used is commercially available, and the graphite/PA66py blends were prepared using the ball milling technique. This technique promotes intercalation since the collisions of the milling spheres reduce particle size, thus producing a more homogeneous mixture.

### 3.5. Thermogravimetric Analyses

**Figure 3:**
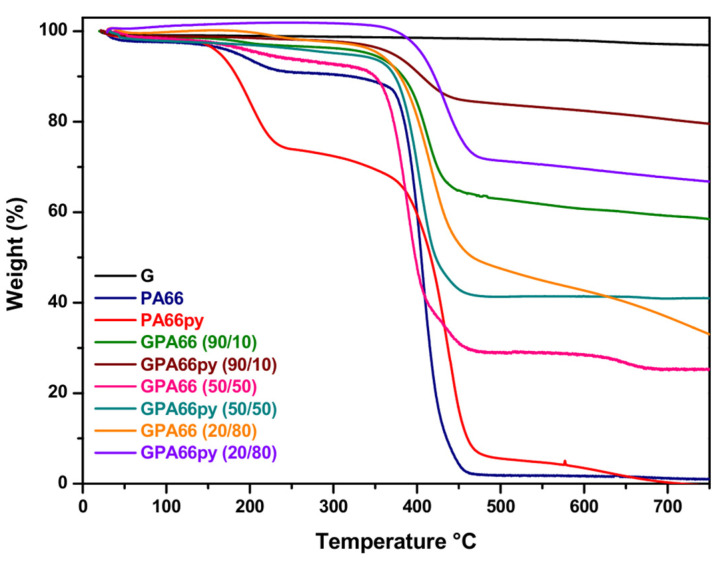
Thermogravimetric curves of graphite G, PA66, PA66py, GPA66, and GPA66py composites at different weight percentage ratios.

Figure 3 displays the thermograms of the flocculent fractions of both the graphite/PA66py samples in the ratios of 90/10, 50/50, and 20/80, along with the graphite/PA66 samples in the same ratios (90/10, 50/50, and 20/80). Furthermore, the thermograms of the graphite used G, polyamide (PA66), and polyamide with pyrene terminal (PA66py) are also reported to facilitate comparison. Graphite shows almost no degradation up to 750 °C, while PA66 and PA66py are completely degraded at 450 °C. Examining the thermogram, it is immediately evident that all the GPA66py samples exhibit a higher residue at 750 °C than GPA66 samples prepared with the same weight ratios. The GPA66py sample made with 80% PA66py and 20% graphite at 750 °C has a residue of 67% ± 3%, whereas the analogous sample made with PA66 and graphite has a residue of only 33% ± 2%.

### 3.6. Differential Scanning Calorimetry

**Figure 4:**
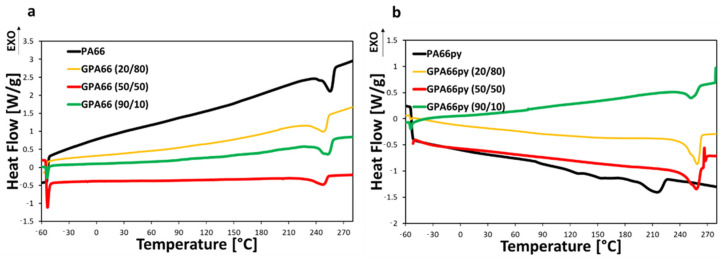
DSC analysis performed on synthesized PA66 composites (**a**) and PA66py composites (**b**).

PA66 and PA66py samples have been thermally characterized to evaluate how the pyrenic chain and the graphite inclusion affect the material’s thermal transitions. In this case, the samples underwent three different steps during the DSC. Firstly, heating from −60 °C to 280 °C, so that residual solvent eventually evaporates, and the material melts completely. Secondly, the material cools down from 280 °C to −60 °C, and then the last heating ramp starts, and the thermal transitions are analyzed. The results of the third heating ramp are reported in Figure 4.

From the evaluation of the DSC, it is evident that PA66py has a more irregular crystalline structure than PA66, with a lower melting point. However, the inclusion of graphite into the PA66py matrix favors the formation of crystalline lattices that melt at higher temperature (up to 259 °C ± 2 °C), as reported in Figure 4b. By contrast, the PA66 melting point decreases from 255 °C ± 1 °C to 247 °C ± 2 °C when graphite is included in the matrix, proving that it affects the crystalline structures that are formed. This result is further confirmed by observing the crystallization curves during the cooling stage. The crystallization peak occurs at higher temperatures for PA66 and especially for PA66py loaded with graphite compared to the unloaded polymer, as reported in Section S2.1 of the Supplementary Materials. This effect is more pronounced on PA66py-loaded samples, probably because of the higher interaction forces between the filler and the sample. This suggests that the filler acts as a nucleating agent, favoring the crystallization of the thermoplastic polymers, in agreement with recent studies [53].

### 3.7. X-Ray Diffraction Measurements

**Figure 5:**
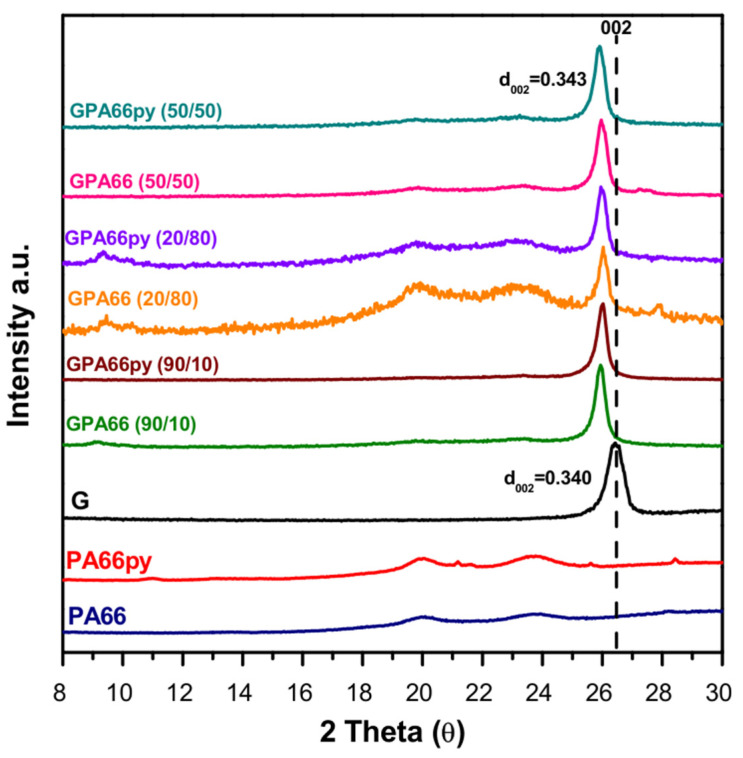
X-Ray diffraction patterns of graphite G, PA66, PA66py, GPA66, and GPA66py composites at different weight percentage ratios.

GPA66 and GPA66py samples, obtained by grinding pristine graphite G with Nylon 6,6 (PA66) and the pyrene-modified one (GPA66py), respectively, in different weight percentage ratios, have also been analyzed through X-ray diffraction spectroscopy (Figure 5).

The X-ray diffraction pattern of the samples at different compositions reveals interesting results related to the characteristic 002 graphitic reflection, which is attributed to the interlayer distance between the graphitic planes (d_002_ = 0.340 nm). Specifically, a slight shift to lower theta becomes more relevant as the percentage of the polymer increases, going from 26.2 (d_002_ = 0.340 nm) in the graphite powder up to 25.9 (d_002_ = 0.343 nm) in the GPA66py in a ratio of 20/80 as well as 50/50, corresponding to an increase in interlayer distance.

This effect is still evident, although less pronounced, in all the other compositions, highlighting a possible intercalation of the polymer chains between the graphitic layers.

Moreover, irrespective of the powder composition, the milling action reduces the half-height width, resulting in an increased correlation length (D) from 13.5 nm to 19.8 nm in most of the samples.

This is an intriguing effect compared to what typically happens to crystalline graphite after ball milling.

The dry grinding treatment of crystalline graphite has been reported to reduce particle size [54,55]. Conversely, some of us noted in a recent paper [52] that ball milling in the presence of Pyren-1-yl-Stearate and graphite in a ratio of 50/50 promoted a double effect of exfoliation and intercalation, resulting in a reduction in half-height width and the aggregation of graphene layers.

The results obtained in the current study show a similar behavior, indicating that the milling action is simultaneously beneficial to the polymer intercalation in graphitic layers, probably due to π-π stacking between pyrene group and graphite planes, as well as the increase in crystallinity, but in contrast to the previous paper, no exfoliation was detected, possibly due differences in the nature and polarity of the polymer chains.

### 3.8. Morphological Analysis

**Figure 6:**
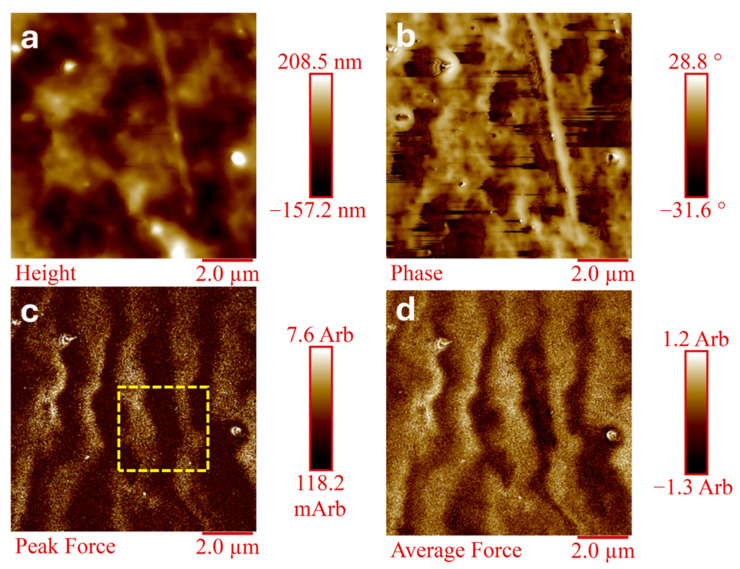
H-AFM images, namely Height (**a**), Phase (**b**), Peak Force (**c**), and Average Force (**d**) of GPA66 (20/80).

**Figure 7:**
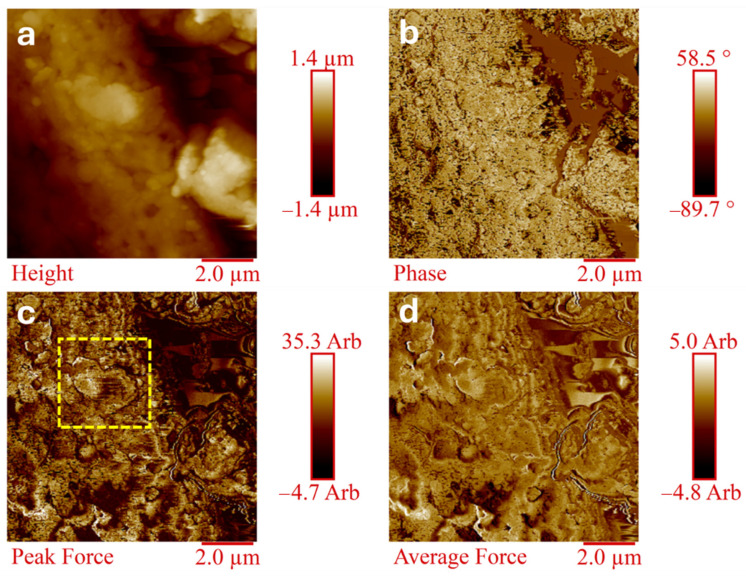
H-AFM images, namely Height (**a**), Phase (**b**), Peak Force (**c**), and Average Force (**d**) of GPA66py (20/80).

AFM analysis is commonly used for characterizing complex polymeric structures [56]. The composites GPA66 (20/80) and GPA66py (20/80) were characterized via H-AFM. The direct morphological comparison between the two samples aims to highlight the compatibilizing effect of the graphitic filler PA66py, containing a pyrene group as a terminal chain in the polyamide (G) chemical structure, together with the interphase properties. In this regard, Figure 6 and Figure 7 show the four H-AFM images (Height, Phase, Peak Force, Average Force) of GPA66 (20/80) and GPA66py (20/80) samples, respectively.

The H-AFM images allow for the discrimination of differences between the two analyzed samples, providing a clear view of the intrinsic morphological characteristics of each, derived from the synergy of information obtainable from the four types of H-AFM images. In particular, the Height image (Figure 7a) of GPA66py (20/80) shows the characteristic flake-like structure of graphite. The Phase image (Figure 7b), particularly useful for distinguishing between areas of different material composition, highlights the homogeneous surface of the composite attributable to the robust interconnections due to π-π stacking interactions at the nanometric level between the graphitic filler PA66py containing a pyrene group as a terminal chain and the polyamide (G), thus confirming the effective compatibility between the polymeric matrix and the filler PA66py, which leads to an improvement in interphase properties. In fact, it is well known that the interphase refers to the region around the filler particles where interactions between the polymer and the filler occur. Improved compatibility ensures better adhesion and interaction at this interface, resulting in strong interfacial bonding.

By contrast, all H-AFM images (Figure 6) of the GPA66 (20/80) composite show that the interphase between the graphitic layer and the polymeric matrix is extremely neat, suggesting a lower compatibility of the graphite G with the filler PA66.

**Figure 8:**
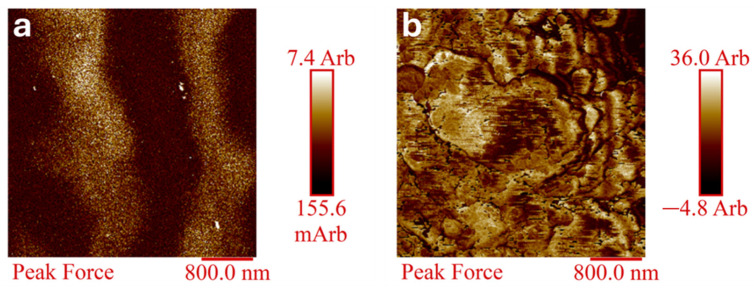
Comparison of the Peak Force images of GPA66 (20/80) (**a**) and GPA66py (20/80) (**b**).

In particular, by comparing the Peak Force images of the two systems GPA66 (20/80) and GPA66py (20/80) (Figure 8) obtained by enlarging the areas highlighted by the yellow dotted rectangle visible in Figure 6 and Figure 7, it is possible to better observe the difference in terms of interphase properties resulting from the compatibilizing effect of the graphitic filler PA66py containing a pyrene group as a terminal chain in the polyamide (G) chemical structure.

For the GPA66 (20/80) system, the difference in properties and in the mechanical response between the graphite and the polymeric matrix is visible, leading to an evident separation in the properties of the two components in the composite material. This phenomenon in composite materials can lead to several disadvantages, including reduced mechanical properties.

On the other hand, for the GPA66py (20/80) system (Figure 8b), the polymeric matrix surrounds the graphitic filler, probably because of the enhanced π-π stacking interactions between the pyrenic chain and the graphite. The materials’ mechanical response appears more homogeneous, resulting in improved mechanical properties in the composite.

**Figure 9:**
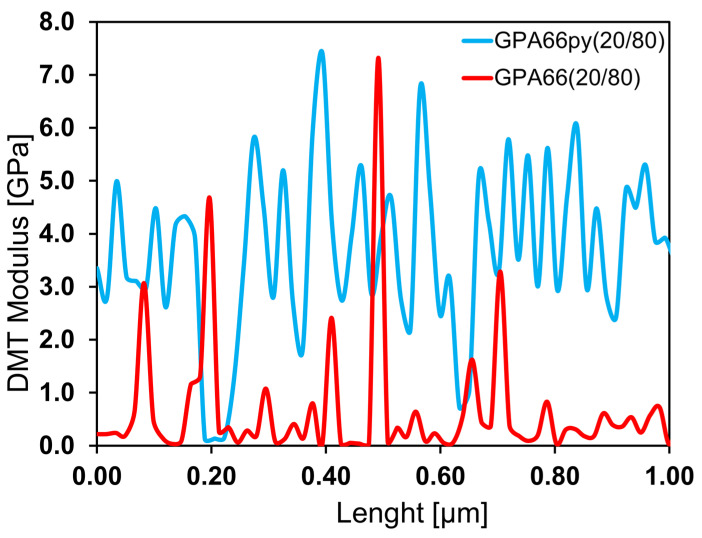
DMT moduli analysis of GPA66 (20/80) and GPA66py (20/80).

The analysis of the DMT moduli between the two systems confirms this hypothesis. In fact, by reporting the local mechanical modulus over the length of the analyzed section (see Figure 9), it is possible to observe that the maximum and minimum values of the mechanical moduli (related to the presence of the polymer and the graphite, respectively) are similar between the two samples. However, in the case of GPA66 (20/80), there are wider regions with a lower mechanical modulus (associated with the presence of the polymer). More precisely, we can observe that the frequency with which the stiffer zones alternate with the softer ones on the analyzed surface is lower in the GPA66 (80/20) sample compared to the GPA66py (80/20) sample. For this reason, the pyrenic chain reasonably guarantees a more efficient intercalation of nylon within the graphitic network, with a consequent higher average mechanical modulus of GPA66py (80/20) compared to GPA66 (80/20).

Overall, the compatibility of the graphitic structure, enhanced by the presence of the pyrenic chain, guarantees higher thermal stability and improved local mechanical performance.

## 4. Conclusions

In the present research, different polyamide polymeric matrices have been synthesized. Nylon 6,6 with pyrenic chain end has been synthesized to increase chemical compatibility with carbonaceous fillers. Composite materials of Nylon 6,6 (with the terminal pyrenic chain) with expanded graphite have been produced via ball milling at various filler concentrations (from 20% up to 90% in the weight of filler). For comparison, composites of Nylon 6,6 without the terminal pyrenic chain have been produced with the same filler content. The structural analysis performed via XRD confirms that the pyrenic chain terminal favors the intercalation of the polymeric chain into the interlayer space within the graphitic block. The thermal properties of the composites have been evaluated using differential scanning calorimetry and thermogravimetric analysis, which revealed differences in the melting peak and, more importantly, in the thermal degradation of the polymer. Probably, because of the polymer intercalation into the graphitic block and the π-π stacking interaction between the aromatic portions, the thermal degradation of the polymer chains is shielded. This phenomenon leads to a significant increase in the residual of the composite at 700 °C (for 20% filler content, the residual increases from 33% to about 67%). The improvement in the local mechanical properties of the composites and the interphase properties suggests that these methodologies could also be exploited for the compatibilization of different types of thermoplastic matrices with various carbonaceous fillers (e.g., carbon nanotubes, graphene, etc.). This could lead to the production of thermoplastic composites with smart functionalities (e.g., high electrical conductivity that can be exploited for activating de-icing or self-sensing functions). Moreover, the relevant enhancement of the thermal and mechanical properties of the Nylon composite leads to a widening of its potential applications in the transportation field (e.g., automotive components, washers, etc.).

## Figures and Tables

**Table 1:** Mixtures of polyamide and graphite after ball milling treatment: flocculent and dusty fractions.

Sample	Flocculent Fraction (%)	Dusty Fraction (%)
GPA66 (90/10)	16	84
GPA66py (90/10)	24	76
GPA66 (50/50)	79	21
GPA66py (50/50)	70	30
GPA66 (20/80)	100	-
GPA66py (20/80)	100	-

## Data Availability

Data are contained within the article.

## Supplementary Materials

The following supporting information can be downloaded at https://www.mdpi.com/article/10.3390/polym17131735/s1, Section S1.1: ^1^HNMR *N*-(6-aminohexyl)-4-(pyren-1-yl)butanamide (A6py); Section S1.2: ^13^CNMR *N*-(6-aminohexyl)-4-(pyren-1-yl)butanamide (A6py); Section S1.3: ^13^CNMR *N*-(6-aminohexyl)-4-(pyren-1-yl)butanamide (A6py); Section S1.4: ^1^HNMR *N*-(6-aminohexyl)-4-(pyren-1-yl)butanamide- Nylon 6,6 (PA66py); Section S1.5: ^13^CNMR *N*-(6-aminohexyl)-4-(pyren-1-yl)butanamide- Nylon 6,6 (PA66py); Section S1.6: ^1^HNMR Nylon 6,6 (PA66); Section S1.7: ^13^CNMR Nylon 6,6 (PA66); Section S2.1 Cooling DSC curves of PA66 and PA66py samples.
