# Supplementary material for: Synthesis of Nylon 6,6 with Pyrene Chain-End for Compatibilization with Graphite and Enhancement of Thermal and Mechanical Properties
Source: Polymers (Basel). 2025 Jun 22;17(13):1735. doi: 10.3390/polym17131735 (PMC12251805; doi:10.3390/polym17131735)

# Synthesis of Nylon 6,6 with Pyrene Chain-End for Compatibilization with Graphite and Enhancement of Thermal and Mechanical Properties

**Veronica Balzano[1], Annaluisa Mariconda[2], Maria Rosaria Acocella[1], Marialuigia Raimondo[3], Assunta D'Amato[1], Pasquale Longo[1], Liberata Guadagno[3], and Raffaele Longo[3,*].**

1 Department of Chemistry and Biology, University of Salerno, Via Giovanni Paolo II, 132, 84084 Fisciano, Italy; vbalzano@unisa.it; macocella@unisa.it; asdamato@unisa.it; plongo@unisa.it;
2 Department of Basic and Applied Sciences, University of Basilicata, Via Dell'Ateneo Lucano 10, 85100 Potenza, Italy; annaluisa.mariconda@unibas.it;
3 Department of Industrial Engineering, University of Salerno, Via Giovanni Paolo II, 132, 84084 Fisciano, Italy; mraimondo@unisa.it; lguadagno@unisa.it; rlongo@unisa.it;
* Correspondence: rlongo@unisa.it;

## INDEX

**S1.1 $^{1}$HNMR *N*-(6-aminohexyl)-4-(pyren-1-yl)butanamide (A6py)** ........ 2
**S1.2 $^{13}$CNMR *N*-(6-aminohexyl)-4-(pyren-1-yl)butanamide (A6py)** ........ 3
**S1.3 $^{13}$CNMR *N*-(6-aminohexyl)-4-(pyren-1-yl)butanamide (A6py)** ........ 4
**S1.4. $^{1}$HNMR *N*-(6-aminohexyl)-4-(pyren-1-yl)butanamide- Nylon 6,6 (PA66py)** ........ 5
**S1.5. $^{13}$CNMR *N*-(6-aminohexyl)-4-(pyren-1-yl)butanamide- Nylon 6,6 (PA66py)** ........ 6
**S1.6. $^{1}$HNMR Nylon 6,6 (PA66)** ........ 7
**S1.7. $^{13}$CNMR Nylon 6,6 (PA66)** ........ 8

## S1.1 $^1$HNMR *N*-(6-aminohexyl)-4-(pyren-1-yl)butanamide (A6py)

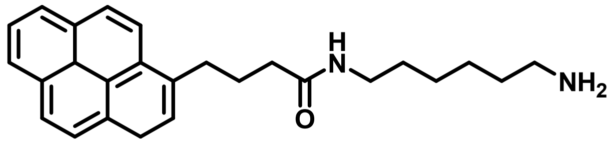


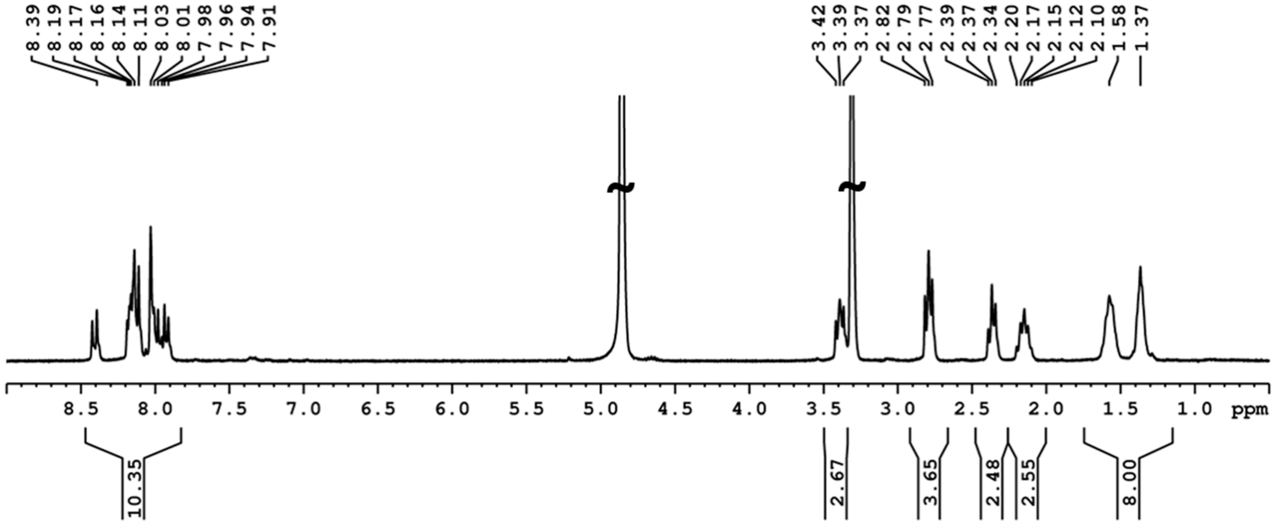


$^1$H NMR (300 MHz, $CD_3OD$) δ: 8.39-7.91- (8H, aromatics); 3.39 (t, 2H, *J* 8.10 Hz, $(C\underline{H_2}(C{=}O)NH)$); 2.79 (t, 4H, *J* 7.63, Hz, $(NH\text{-}C\underline{H_2}\text{-}(CH_2)_4\text{-}C\underline{H_2}\text{-}NH_2)$); 2.37 (t, 2H, *J* 7.29 Hz, $(Ar\text{-}C\underline{H_2})$); 2.15 (m, 2H, $(Ar\text{-}CH_2\text{-}C\underline{H_2})$); 1.58-1.37 (br s, 8H, $(NH\text{-}CH_2\text{-}(C\underline{H_2})_4\text{-}CH_2\text{-}NH_2)$).

## S1.2 $^{13}$CNMR *N*-(6-aminohexyl)-4-(pyren-1-yl)butanamide (A6py)

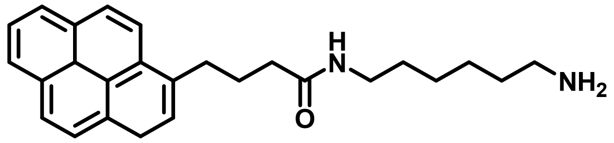


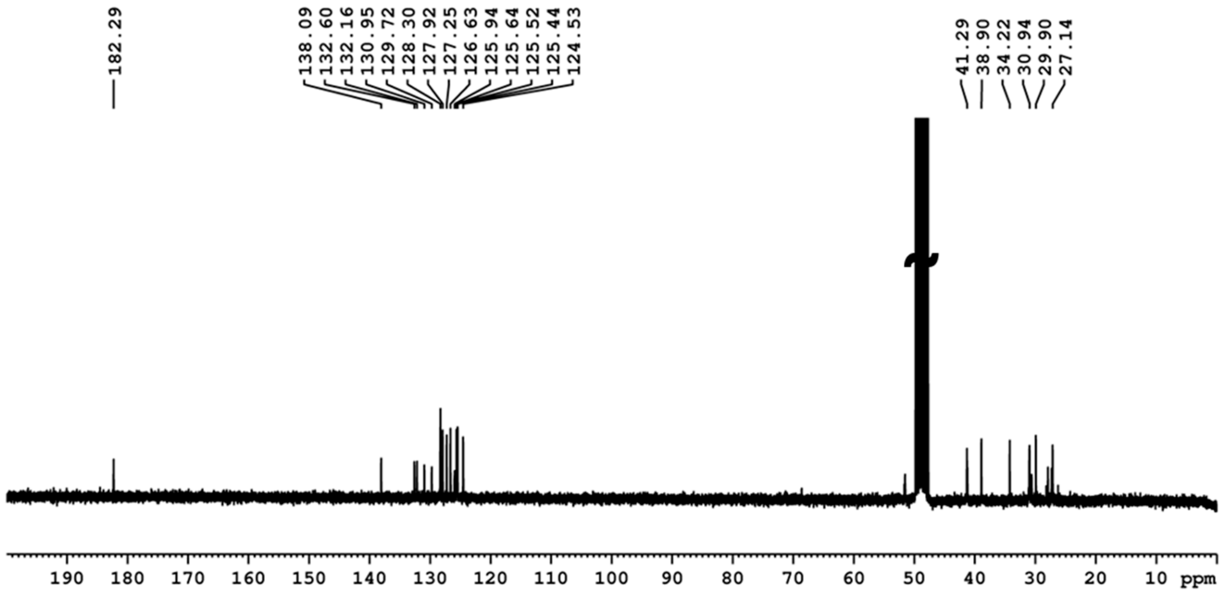


$^{13}$CNMR (75 MHz, $CD_3OD$) δ: 182.2$_9$ ($CH_2$($\underline{C}$=O)NH); 138.0$_9$ -124.5$_3$ (aromatics); 41.2$_9$ (NH-$\underline{C}H_2$-$CH_2$-$(CH_2)_2$-$CH_2$-$\underline{C}H_2$-$NH_2$); 38.9$_0$ ($\underline{C}H_2$(C=O)NH); 34.2$_2$ (Ar-$\underline{C}H_2$); 30.9$_4$ (NH-$CH_2$-$\underline{C}H_2$-$(CH_2)_2$-$\underline{C}H_2$-$CH_2$-$NH_2$); 29.9$_0$ (NH-$CH_2$-$CH_2$-($\underline{C}H_2$)$_2$-$CH_2$-$CH_2$-$NH_2$); 27.1$_4$ (Ar-$CH_2$-$\underline{C}H_2$).

**S1.3 $^{13}$CNMR *N*-(6-aminohexyl)-4-(pyren-1-yl)butanamide (A6py)**

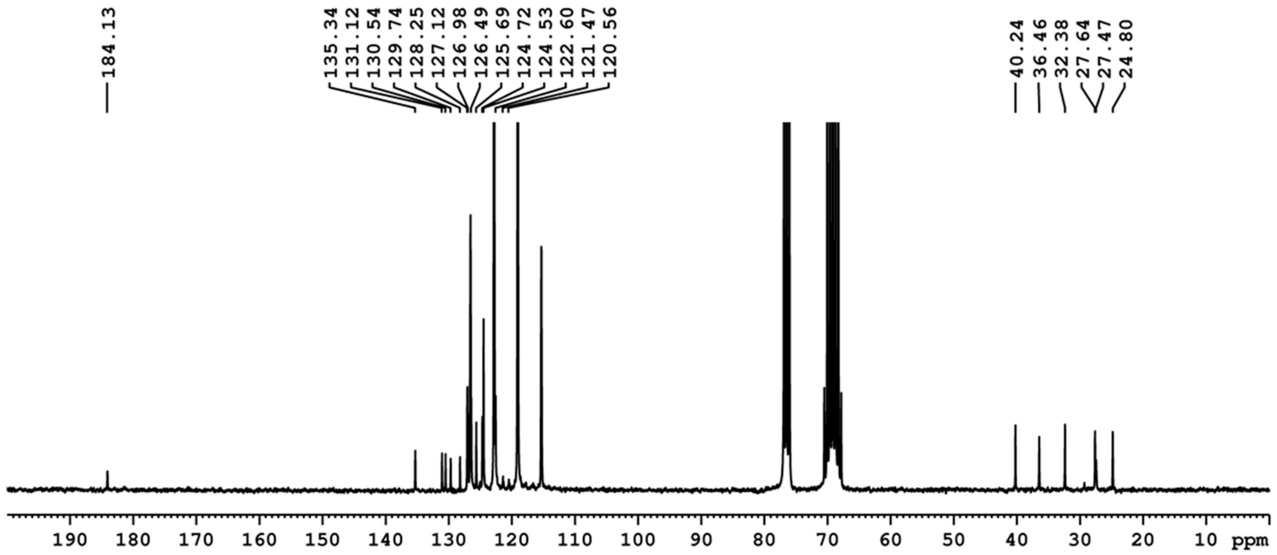


$^{13}$CNMR (75 MHz, HFP/$CDCl_3$) δ: 184.1$_3$ ($CH_2$($\underline{C}$=O)NH); 135.3$_4$ -120.5$_6$ (aromatics); 40.2$_4$ (NH-$\underline{C}H_2$-$CH_2$-$(CH_2)_2$-$CH_2$-$\underline{C}H_2$-$NH_2$); 36.4$_6$ ($\underline{C}H_2$(C=O)NH); 32.3$_8$ (Ar-$\underline{C}H_2$); 27.6$_4$ (NH-$CH_2$-$\underline{C}H_2$-$(CH_2)_2$-$\underline{C}H_2$-$CH_2$-$NH_2$); 27.4$_7$ (NH-$CH_2$-$CH_2$-$(\underline{C}H_2)_2$-$CH_2$-$CH_2$-$NH_2$); 24.8$_0$ (Ar-$CH_2$-$\underline{C}H_2$).

## S1.4. $^{1}$HNMR *N*-(6-aminohexyl)-4-(pyren-1-yl)butanamide- Nylon 6,6 (PA66py)

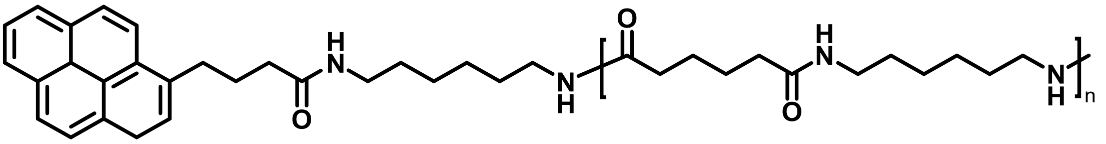

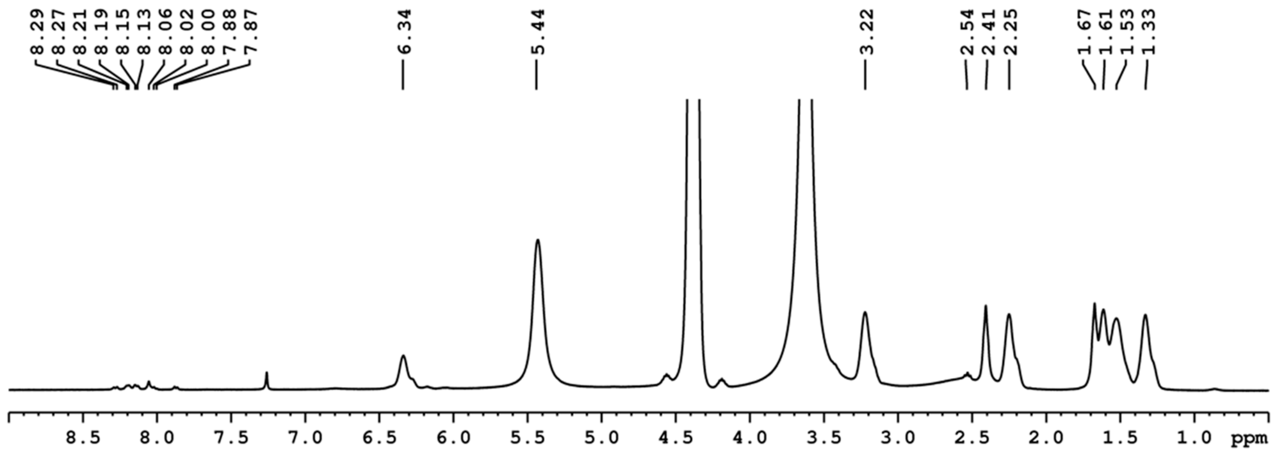


$^{1}$H NMR: (400 MHz, HFP/$CDCl_3$) δ: 8.29-7.87 (aromatics); 6.34 (br, (-N$\underline{\mathit{H_2}}$)); 5.44 (br, (-N$\underline{\mathit{H}}$-$CH_2$)); 3.22 (br s, overlapping, 6H, ((C=O)C$\underline{\mathit{H_2}}$-$(CH_2)_2$-C$\underline{\mathit{H_2}}$(C=O)), (C$\underline{\mathit{H_2}}$(C=O))); 2.54 (br s, overlapping, 2H, (Ar-C$\underline{\mathit{H_2}}$)); 2.41(br s, overlapping, 6H, ((C=O)$CH_2$-(C$\underline{\mathit{H_2}}$$)_2$-$CH_2$(C=O)); 2.25, (br s, overlapping, 8H, ((NH-C$\underline{\mathit{H_2}}$-$(CH_2)_4$-C$\underline{\mathit{H_2}}$-$NH_2$)); 1.67-1.33 (br s, overlapping, 16H, (NH-$CH_2$-(C$\underline{\mathit{H_2}}$$)_4$-$CH_2$-$NH_2$)).

## S1.5. $^{13}$CNMR *N*-(6-aminohexyl)-4-(pyren-1-yl)butanamide- Nylon 6,6 (PA66py)

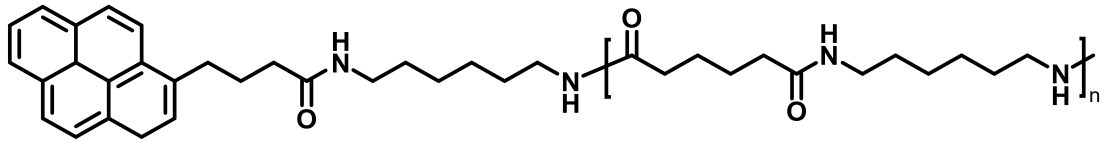

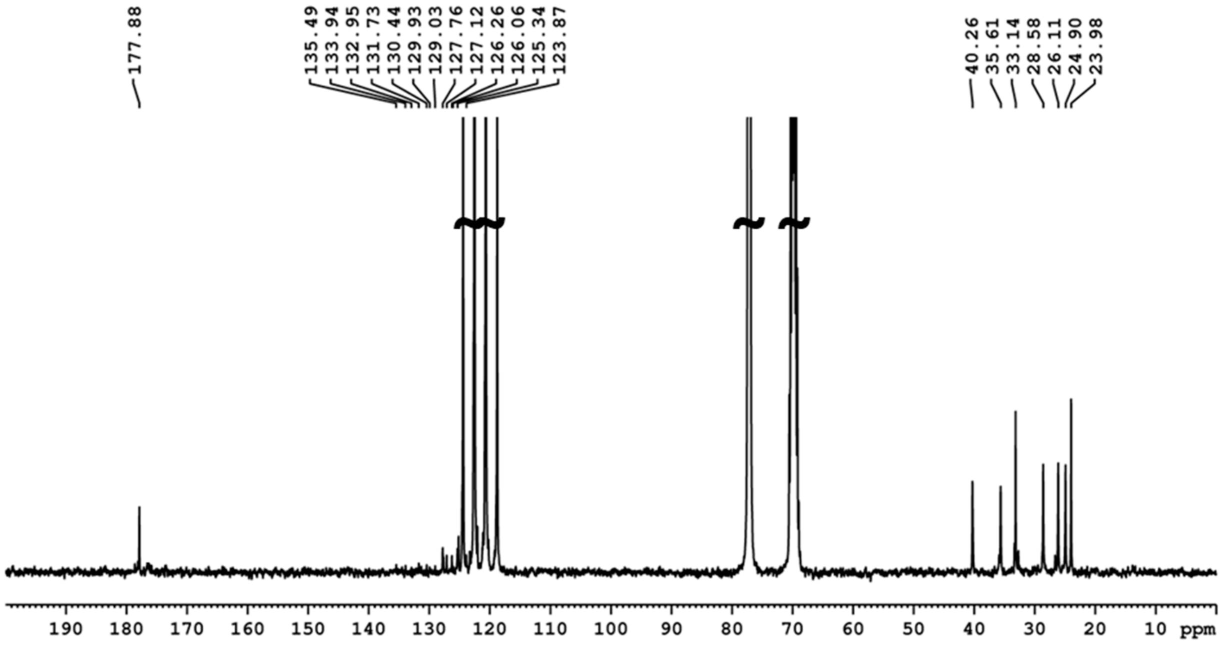


$^{13}$CNMR: (150 MHz, HFP/$CDCl_3$) δ: $177.8_8$ ($CH_2$(C=O)NH); $135.4_9$ -$123.8_7$ (aromatics); $40.2_6$ (NH-$CH_2$-$CH_2$-$(CH_2)_2$-$CH_2$-$CH_2$-$NH_2$); $33.1_4$ (Ar-$CH_2$); $35.6_1$ ($CH_2$(C=O)NH, (C=O)$CH_2$-$(CH_2)_2$-$CH_2$(C=O)); $28.5_8$ (NH-$CH_2$-$CH_2$-$(CH_2)_2$-$CH_2$-$CH_2$-$NH_2$); $26.1_1$ (NH-$CH_2$-$CH_2$-$(CH_2)_2$-$CH_2$-$CH_2$-$NH_2$); $24.9_0$ (C=O)$CH_2$-$(CH_2)_2$-$CH_2$(C=O); $23.9_8$ (Ar-$CH_2$-$CH_2$).

## S1.6. $^{1}$HNMR Nylon 6,6 (PA66)

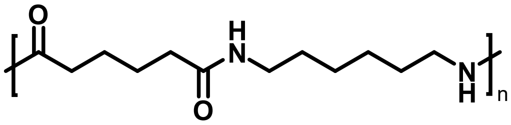


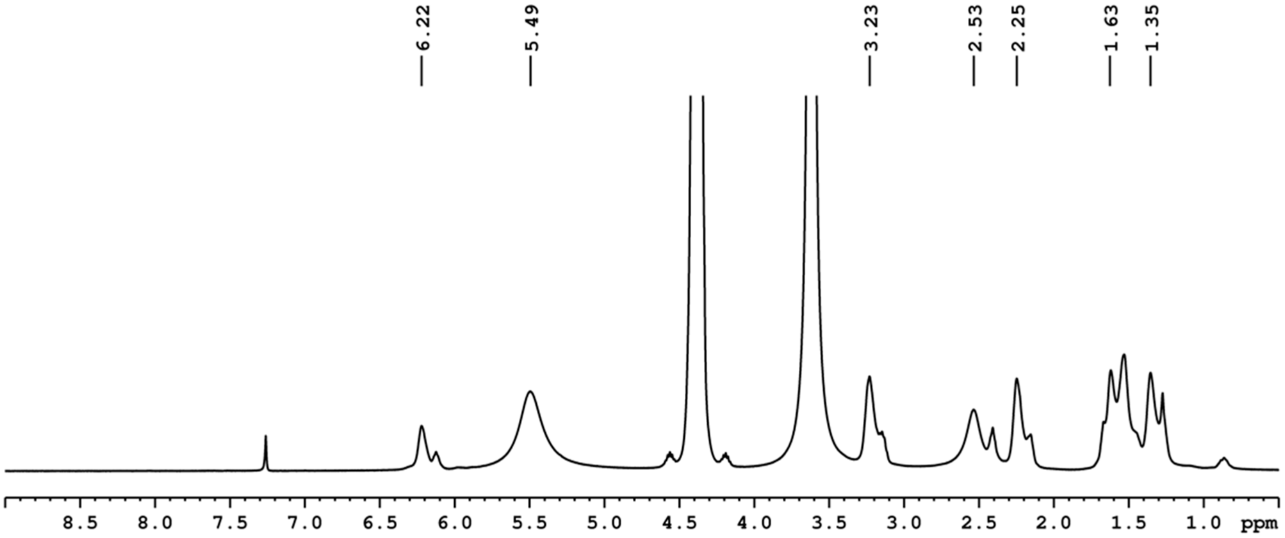


$^{1}$H NMR: (400 MHz, HFP/$CDCl_3$) δ: 6.22 (br, (-N$\underline{H_2}$)); 5.49 (br, (-N$\underline{H}$-$CH_2$)); 3.23 (br, s, overlapping, 4H, (C=O)C$\underline{H_2}$-$(CH_2)_2$-C$\underline{H_2}$(C=O)); 2.53 (br, s, overlapping, 4H, (NH-C$\underline{H_2}$-$(CH_2)_4$-C$\underline{H_2}$-$NH_2$)); 2.25 (br, s, overlapping, 4H, (C=O)$CH_2$-(C$\underline{H_2}$)$_2$-$CH_2$(C=O)); 1.63-1.35 (br, s, overlapping, 8H, (NH-$CH_2$-(C$\underline{H_2}$)$_4$-$CH_2$-$NH_2$)).

## S1.7. $^{13}$CNMR Nylon 6,6 (PA66)

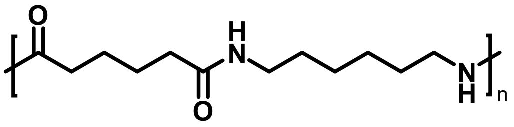

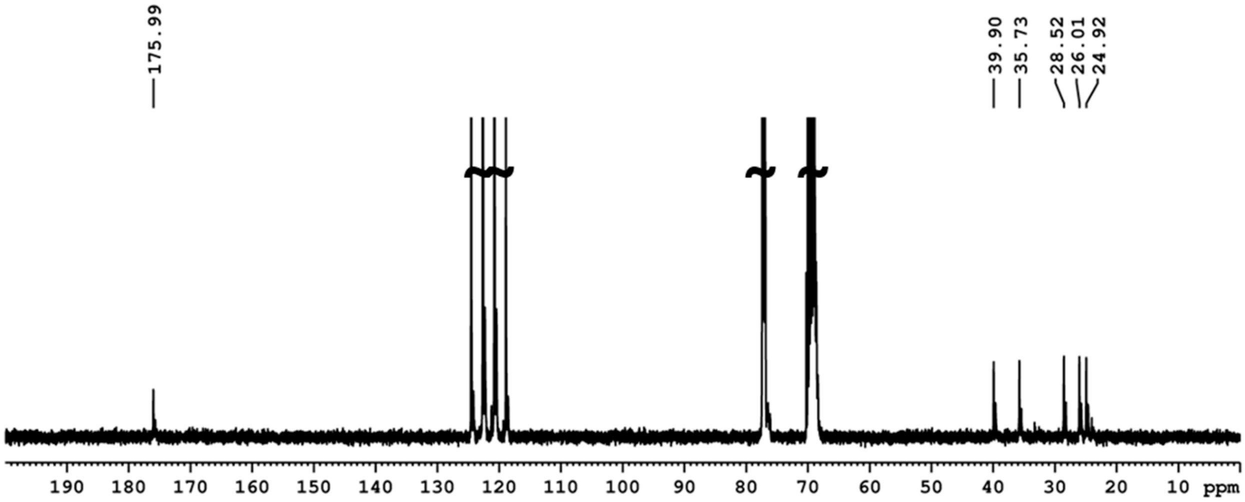


$^{13}$CNMR:(150 MHz, HFP/$CDCl_3$) δ: $175.9_9$ ($CH_2(\underline{C}{=}O)NH$); $39.9_0$ (NH-$\underline{C}H_2$-$CH_2$-$(CH_2)_2$-$CH_2$-$\underline{C}H_2$-$NH_2$); $35.7_3$ ($\underline{C}H_2(C{=}O)NH$); $28.5_2$ (NH-$CH_2$-$\underline{C}H_2$-$(CH_2)_2$-$\underline{C}H_2$-$CH_2$-$NH_2$); $26.0_1$ (NH-$CH_2$-$CH_2$-$(\underline{C}H_2)_2$-$CH_2$-$CH_2$-$NH_2$); $24.9_2$ (C=O)$CH_2$-$(\underline{C}H_2)_2$-$CH_2$(C=O).

## S2.1. Cooling DSC curves of PA66 and PA66py samples

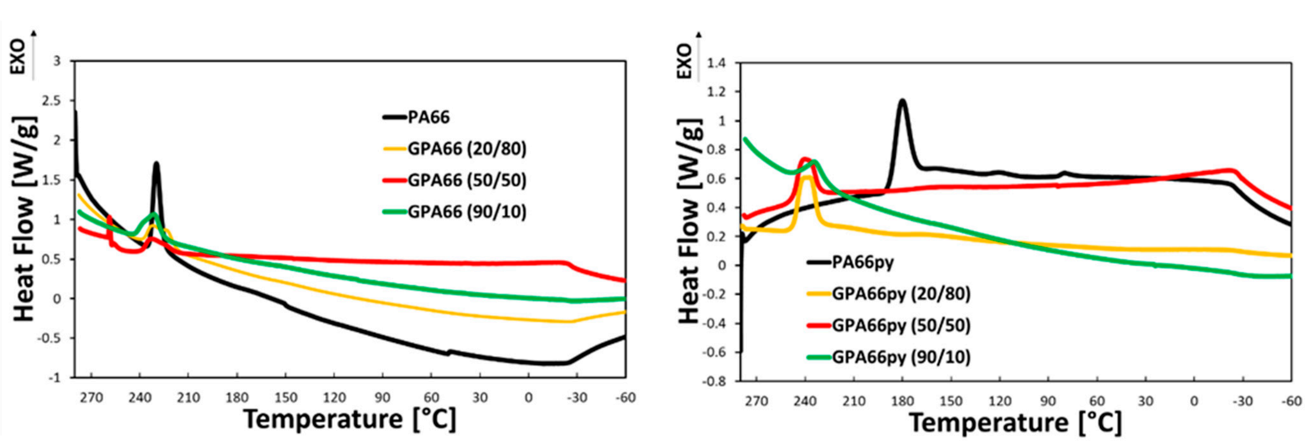

Supplement: Supplementary file 1 [file polymers-17-01735-s001.zip › polymers-3676243-supplementary.pdf]
